# Microbiota Modulate Anxiety-Like Behavior and Endocrine Abnormalities in Hypothalamic-Pituitary-Adrenal Axis

**DOI:** 10.3389/fcimb.2017.00489

**Published:** 2017-11-30

**Authors:** Ran Huo, Benhua Zeng, Li Zeng, Ke Cheng, Bo Li, Yuanyuan Luo, Haiyang Wang, Chanjuan Zhou, Liang Fang, Wenxia Li, Rong Niu, Hong Wei, Peng Xie

**Affiliations:** ^1^Department of Neurology, Yongchuan Hospital, Chongqing Medical University, Chongqing, China; ^2^Institute of Neuroscience and the Collaborative Innovation Center for Brain Science, Chongqing Medical University, Chongqing, China; ^3^Key Laboratory of Clinical Laboratory Diagnostics (Ministry of Education), Department of Laboratory Medicine, Chongqing Medical University, Chongqing, China; ^4^Department of Laboratory Animal Science, College of Basic Medical Sciences, Third Military Medical University, Chongqing, China; ^5^Department of Neurology, First Affiliated Hospital of Chongqing Medical University, Chongqing Medical University, Chongqing, China

**Keywords:** intestinal microbes, HPA axis, CRS model, microbiota-gut-brain axis, stress-related diseases

## Abstract

Intestinal microbes are an important system in the human body, with significant effects on behavior. An increasing body of research indicates that intestinal microbes affect brain function and neurogenesis, including sensitivity to stress. To investigate the effects of microbial colonization on behavior, we examined behavioral changes associated with hormones and hormone receptors in the hypothalamic-pituitary-adrenal (HPA) axis under stress. We tested germ-free (GF) mice and specific pathogen-free (SPF) mice, divided into four groups. A chronic restraint stress (CRS) protocol was utilized to induce external pressure in two stress groups by restraining mice in a conical centrifuge tube for 4 h per day for 21 days. After CRS, Initially, GF restraint-stressed mice explored more time than SPF restraint-stressed mice in the center and total distance of the OFT. Moreover, the CRH, ACTH, CORT, and ALD levels in HPA axis of GF restraint-stressed mice exhibited a significantly greater increase than those of SPF restraint-stressed mice. Finally, the Crhr1 mRNA levels of GF CRS mice were increased compared with SPF CRS mice. However, the Nr3c2 mRNA levels of GF CRS mice were decreased compared with SPF CRS mice. All results revealed that SPF mice exhibited more anxiety-like behavior than GF mice under the same external stress. Moreover, we also found that GF mice exhibited significant differences in, hormones, and hormone receptors compared with SPF mice. In conclusion, Imbalances of the HPA axis caused by intestinal microbes could affect the neuroendocrine system in the brain, resulting in an anxiety-like behavioral phenotype. This study suggested that intervention into intestinal microflora may provide a new approach for treating stress-related diseases.

## Introduction

The intestine is the largest system in the mammalian body, containing 100 trillion organisms. Intestinal microbial flora are established in early life in mammals, and affect the host's physiological function (Grenham et al., 2011; Lozupone et al., 2012; Heitlinger et al., 2017). Recent studies also have reported that intestinal microbial steady-state imbalances can cause a range of metabolic diseases (Wen et al., 2008; Henaomejia et al., 2012; Koren et al., 2012). A number of studies have explored the mechanisms of intestinal microorganisms, and a range of microbe-related diseases have been discovered and explored in neuropsychiatric subjects. However, the precise mechanisms of action of intestinal microbial flora remain unclear. Among the known pathogenetic mechanisms, several mental illnesses have been linked to the hypothalamic-pituitary-adrenal (HPA) axis (Schatzberg et al., 2014; Fries et al., 2015).

According to the long-standing HPA axis imbalance theory, hormone imbalance is closely associated with psychiatric diseases. A range of factors, including exercise, anxiolytic drugs, and sexual experience, can interfere with the secretion of stress hormones related to the HPA axis (Romero, 2004). Meanwhile, stress-related psychiatric disorders are closely related to imbalances in the HPA axis (Jacobson, 2014; van Bodegom et al., 2017), including anxiety disorders, social anxiety disorder, and post-traumatic stress disorder (Wirtz et al., 2007). Several studies have reported that changes in HPA axis hormones vary between stimulus type and rat variety, and can be used as an index of the intensity of a stressor (Girotti et al., 2006). In addition, one study found that plasma hormone levels (adrenocorticotropic hormone, ACTH; cortisol, CORT) were increased in the HPA axis after exposure to various stressors for 30 min (Hueston et al., 2011) and decreased to baseline levels within a certain time after the termination of acute stress (Dhabhar et al., 1997). The glucocorticoid receptor (GR) and the mineralocorticoid receptor (MR) mediate regulation of CORT gene expression (Arriza et al., 1987), which illustrates that hormonal changes in the HPA axis may are associated with changes in receptor levels. Interestingly, previous studies have found microbes are closely connection between HPA axis and behavior (Moya-Pérez et al., 2017).

In recent studies, GF mice are widely used as a tool for assessing the role of intestinal microbes, which have been found to affect mouse brain function and behavior (Luczynski et al., 2016). In addition, an increasing body of research has examined the effects of intestinal microbes in the HPA axis and microbiota-gut-brain axis using GF animals and antibiotic intervention (Foster, 2015; Zeng et al., 2016). Studies in which stool is transplanted from patients into the intestine of germ-free (GF) mice have revealed that gut microbiota can affect animals' behavior through the microbiota-gut-brain axis (Bercik et al., 2011; Cryan and Dinan, 2012; Zheng et al., 2016b). In the HPA axis, the hypothalamus is considered the starting point of the HPA axis, and previous studies have shown that levels of hormone concentration and hormone receptors in this brain region are altered under acute pressure (Crumeyrolle-Arias et al., 2014; Zhu et al., 2014). To create artificial chronic stress, the chronic restraint stress (CRS) model is classical and widely used to induce external pressure to detect the relationship between chronic pressure and diseases (Andrus et al., 2012). On the basis of this previous research, we hypothesized that intestinal microbial stabilization disorders would affect behavioral changes through the HPA axis using the CRS model in mice.

In the current study, to assess the effects of intestinal microbes on the HPA axis, we first examined behavior, hormone levels and receptor expression in the HPA axis using the CRS model in both GF and SPF mice. Then behavior was analyzed to assess whether differences in intestinal microbes play an important role in behavioral changes in mice.

## Materials and methods

### Animals

GF Kunming (KM) and SPF KM mice (male; 6 weeks old) were provided by the Experimental Animal Center of the Third Military Medical University (Chongqing, China) and bred at the Experimental Animal Center of the Third Military Medical University (GB 14922.2-2011). GF mice were kept and subjected to the CRS protocol in sterile isolators until the beginning of the behavioral tests. Weekly fecal samples were collected from GF mice and monitored using cultures of aerobic and anaerobic microbes to ensure the reliability of sterile feeding conditions. SPF mice were kept and subjected to the CRS protocol in barrier system with 10,000 cleanliness level and noise ≤60 dB. All animals were group-housed in Macrolon cages (37 cm long, 26 cm wide, 17 cm high) and fed with autoclaved chow and water. Animal room conditions were maintained with a constant temperature of 22 ± 2°C, relative humidity 55 ± 5% under a 12 h light-12 h dark cycles (lights on at 8:00 a.m.). The experimental protocols were in accord with the National Institutes of Health Guide for the Care and Use of Laboratory Animals (NIH Publication No. 80-23), revised in 1996. Moreover, the Ethics Committee of Chongqing Medical University approved all the experiments.

### Chronic restraint stress (CRS) procedure

**Figure 1:**
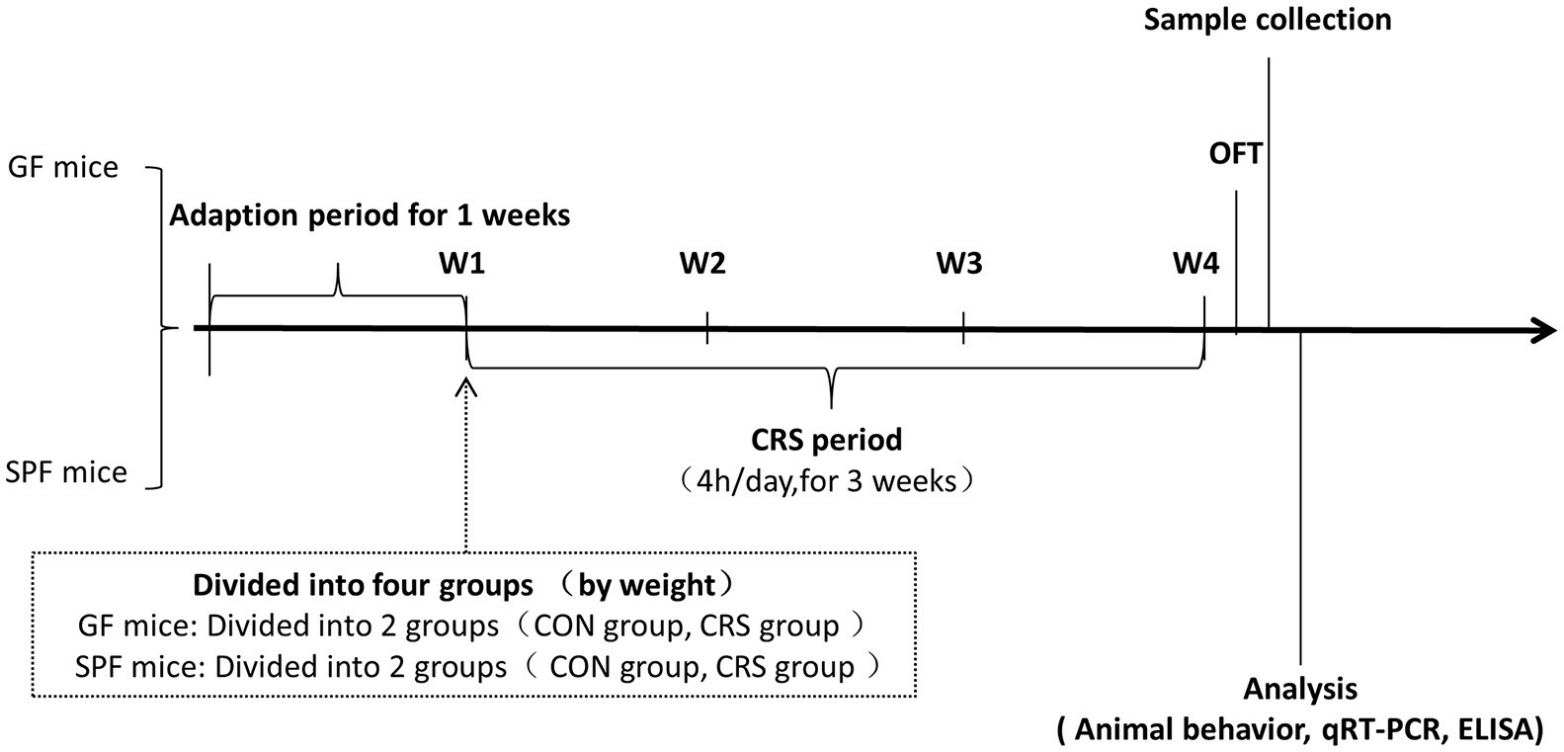
Animal treatment and experimental procedure. W1, animal adaptation time; Animals were grouped on the last day of the first week; W1–W4, CRS period. GF mice, germ-free mice; SPF mice, specific pathogen-free mice; CRS, chronic restraint stress; OFT, open field test; qRT-PCR, quantitative real-time PCR; ELISA, enzyme-linked immunosorbent assay.

All mice were acclimatized to the standard experimental environment for 7 days before the test session (Liu et al., 2016). GF and SPF mice were subjected to an established chronic physical restraint protocol. They were placed in the 50 ml multiple breathable hollows (0.5 mm diameter, 12 holes) conical centrifuge tubes (Wong et al., 2016). This restraint vessel was adapted to the animal's body size, and no pain was involved. Mice were restrained in the pipe for 4 h (from 13:00 to 17:00), with 20 h of rest time each day for 21 days. Mice were deprived of food and water during restraint then given food and water after each restraint experiment (Zafir and Banu, 2007). Mice were released into the cage to receive water and food immediately after the experiment. This restraint procedure was approved by the Ethics Committee of Chongqing Medical University. The details of the experimental procedure are shown in Figure 1.

### Behavioral procedures

In each experiment, GF and SPF mice (*n* = 28–32 in each group) were removed from the bacteria isolator, and placed in the experimental environment for at least 1 h. The whole experimental environment was insulated to maintain a temperature of 22 ± 2°C, and humidity of 55 ± 5%. The trajectory of each mouse was recorded with a video tracking system linked to a computer. Trajectories were analyzed and quantified using the SMART2.5 software package (Panlab, Barcelona, Spain).

### Open field test (OFT)

Mice were gently placed in the center of the apparatus and allowed to move freely. The device was constructed from opaque black paper (45 × 45 × 45 cm), and had no distinctive odor. The position placing each mouse was the fixed edge of the device. After each test, 70% ethanol was utilized to clean feces and remove odor. The test time was 6 min: 1 min to adapt, and 5 min for testing. The whole experimental process was recorded with a video tracking system. Correlative indices were measured in the last 5 min (Kim et al., 2012; Zhang et al., 2016; Zhou et al., 2016).

### Sample collection and preparation

After the experimental period, mice were euthanized with 10% chloral hydrate (400 mg/kg; Chen et al., 2015). Mice were perfused with ice physiological saline (0.9% NaCl, Nongfu Spring Company Limited, Hangzhou, China). The whole brain was dissected and immediately placed in liquid nitrogen. All tissue samples were stored in a refrigerator at −80°C (Wang et al., 2016).

### Hormonal measurement

To quantify changes in HPA axis hormones in the hypothalamus tissue, the concentrations of ACTH, corticotropin-releasing hormone (CRH), CORT, and aldosterone (ALD) were analyzed using an enzyme-linked immunosorbent (ELISA) kit (ACTH, least detectable dose, 0.22 pg/ml, percent coefficient of variation, 5.38%, MD Bioproducts, USA; CRH, least detectable dose, 0.19 ng/ml, percent coefficient of variation, 6.54%; CORT, least detectable dose, 0.19 ng/ml, percent coefficient of variation, 6.66%; ALD, least detectable dose, 18.75 pg/ml, percent coefficient of variation, 4.73%; Elabscience Biotechnology Co., Ltd. China). Hypothalamus tissue was weighed, then minced into small pieces, which were homogenized in 1 g: 9 ml phosphate-buffered saline (PBS; Hyclone Co., USA) with protease inhibitor (Roche, Germany). We allowed samples and reagents to equilibrate to room temperature (22–25°C) before performing the assay. Each procedure was carried out according to the kit instructions, on ice.

Hormonal concentrations from each sample were calculated from the standard curve using CurveExpert 1.30 software (Daniel G. Hyams Co., USA) in accordance with the manufacturer's recommendations and normalized for hypothalamus tissue homogenate protein measured with the BCA method using enhanced BCA protein assay kit (Beyotime Co., China). The ELISA reaction was recorded at the corresponding wavelength using a microplate reader (Bio-Rad Co., USA).

### Hormone-related receptor measurement

Total RNA was extracted from frozen hypothalamus tissue using an RNA mini kit (Ambion, USA) on ice. All experimental procedures were in accord with the kit instructions, followed by reverse-transcribed into DNA using a PrimeScript RT Reagent Kit (Takara, Toyoto, Japan). The cycling conditions were: three cycles of reverse transcription reaction at 37°C for 15 min and reverse transcriptase inactivation reaction at 85°C for 5 s. The mRNA values in the hypothalamus were quantified using qRT-PCR (Roche, Germany). The cycling conditions were: 10 min preincubation at 95°C and 40 cycles of DNA amplification at 95°C for 10 s, 60°C for 30 s, and 72°C for 35 s. Primer sequences were acquired using Primerbank (Harvard, USA), and synthesized by a biotechnology company (Sangon Biotech, Shanghai, China). The primer sequences for Crhr1fwd were as follows: 5′-gggcagcccgtgtgaattatt-3′, rev: 5′-atgacggcaatgtggtagtgc-3′; for Crhr2fwd:5′-catccaccacgtccgagac-3′, rev:5′-ctcgccaggattgacaaagaa-3′; for Mc2rfwd:5′-acaccgcaagaaataactccg-3′, rev:5′-aggaggacaatcaagttctcca-3′; for Nr3c1fwd:5′-agctccccctggtagagac-3′, rev:5′-ggtgaagacgcagaaaccttg-3′; for Nr3c2fwd:5′-gaagagcccctctgtttgcag-3′, rev:5′-tccttgagtgatgggactgtg-3′; for Gapdhfwd:5′-AGGTCGGTGTGAACGGATTTG-3′, rev:5′-TGTAGACCATGTAGTTGAGGTCA-3′. The corresponding mRNA content was standardized with Gapdh mRNA, and data expression was normalized with respect to the corresponding control group. All data were quantified with LightCycler 96 SW 1.1 analysis software (Roche, Germany). Hormonal receptor levels (Crhr1, Crhr2, Mc2r, Nr3c1, Nr3c2) were analyzed using quantitative real-time polymerase chain reaction (qRT-PCR; Roche, Germany) assay.

### Statistical analysis

All data were calculated as single data points superimposed to boxplots. The ELISA data, PCR data, and behavioral data were analyzed using two-way analysis of variance (ANOVA) assay with SPSS 20.0 (IBM North America, New York, NY, USA). In all cases, *p* < 0.05 were considered statistically significant.

## Results

### Changes in behaviors between GF and SPF mice

**Figure 2:**
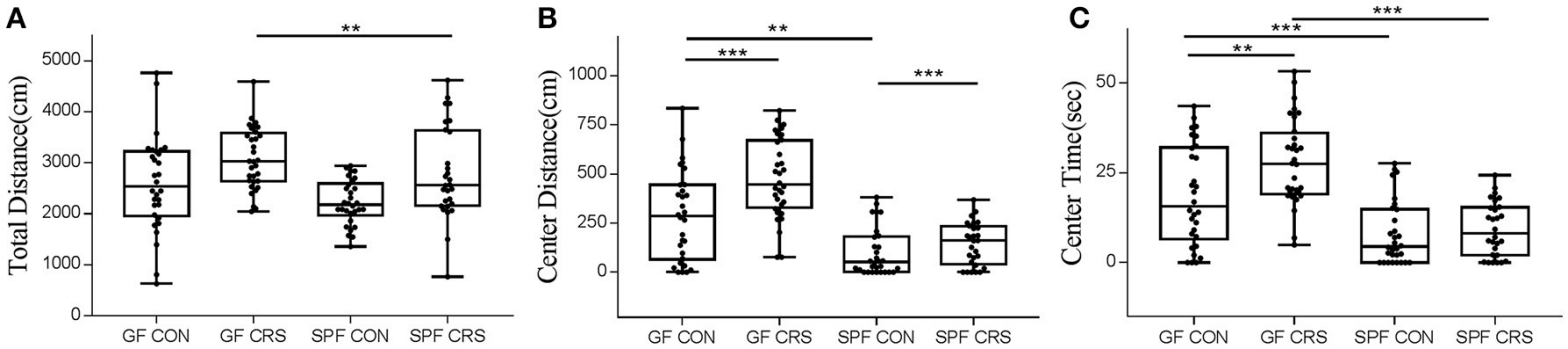
Differences in open field test performance between groups. In the open field test (OFT): total distance (locomotor activity) traveled [**A**, *F*_(3, 116)_ = 4.961], distance in central activity [**B**, *F*_(3, 116)_ = 7.949] and time spent in the center [**C**, *F*_(3, 116)_ = 5.658] for GF CRS mice and SPF CRS mice were compared with the naive group. All values were presented as single data points superimposed to boxplots (*n* = 28–32 in each group). ^**^p < 0.01, ^***^p < 0.001.

In order to determine whether the microbial colonization can alter behavior in mice, the OFT was used to assess behavior. Two-way ANOVA revealed that the SPF stressed control group moved a shorter total distance in the OFT compared with the GF stressed group (*p* < 0.01). Other groups did not show significant differences (Figure 2A). GF non-stressed control animals moved a significantly greater distance than SPF non-stressed mice in the center of the OFT (*p* < 0.01), and GF stressed animals moved a greater distance than GF non-stressed mice (*p* < 0.001). Mice in the SPF CRS group moved a greater distance in the center than those in the SPF non-CRS group (*p* < 0.001; Figure 2B). GF control mice spent less time exploring than GF stressed animals (*p* < 0.01) in the center of the OFT. In addition, the results revealed that GF control mice spent more time exploring than SPF control animals (*p* < 0.001), and GF restraint-stressed mice explored more time than SPF restraint-stressed mice (*p* < 0.001) in the center of the OFT (Figure 2C).

### Hormonal dysfunction of the HPA axis

**Figure 3:**
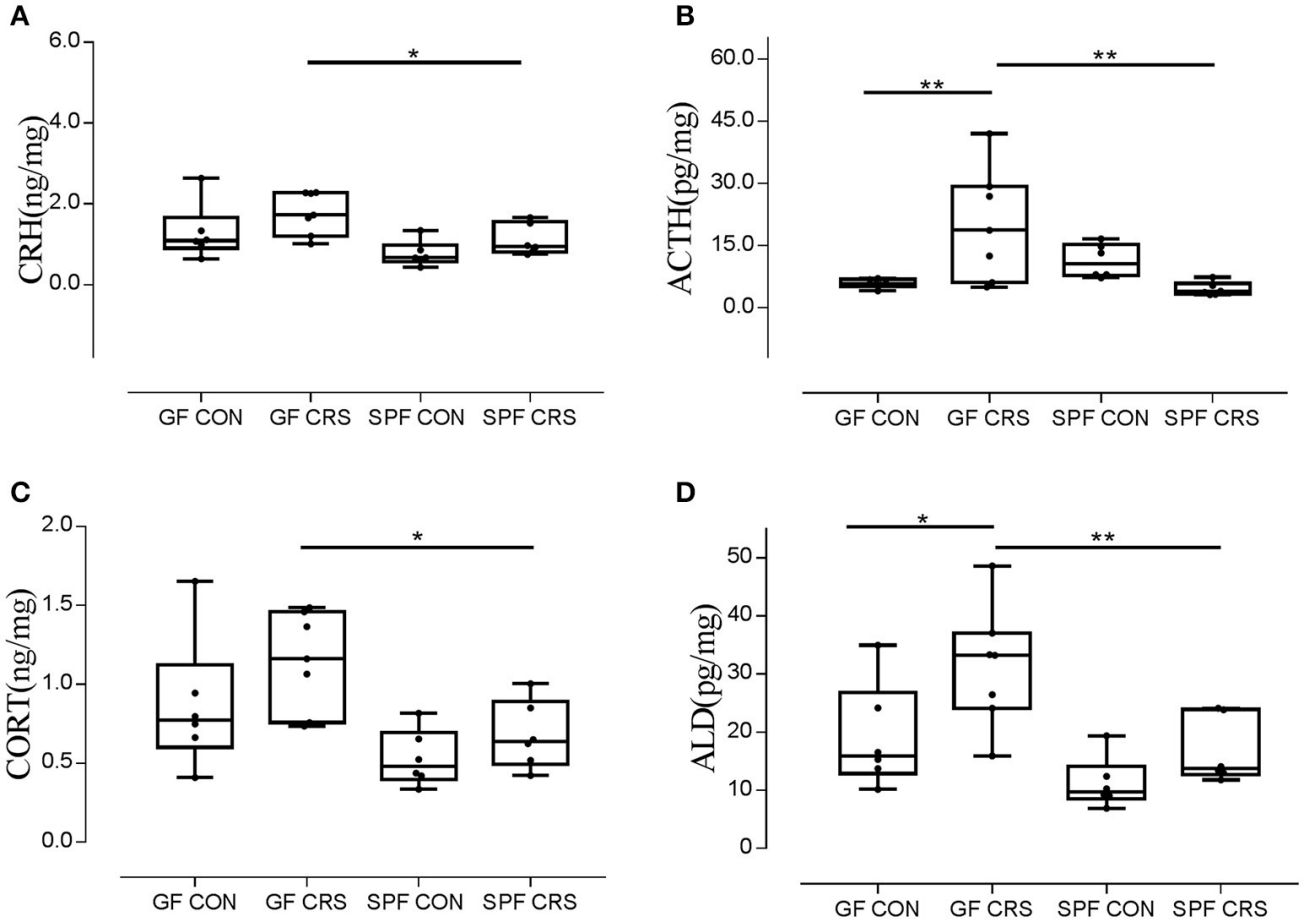
Ratios of CRH, ACTH, CORT, and ALD levels relative to protein in hypothalamus tissue homogenates in control group and CRS group. (*n* = 6–7 in each group). The concentrations of CRH, ACTH, CORT, and ALD in tissue homogenates were measured with an ELISA kit. The concentrations of CRH, ACTH, CORT, and ALD were calculated as ng or pg/mg, hypothalamus protein homogenates. The values were presented as single data points superimposed to boxplots. [**A**, *F*_(3, 21)_ = 0.794; **B**, *F*_(3, 21)_ = 6.197; **C**, *F*_(3, 21)_ = 0.990; **D**, *F*_(3, 21)_ = 1.450]. ^*^p < 0.05, ^**^p < 0.01.

We examined hormone and receptor levels in hypothalamic tissue. To assess changes in HPA axis-related hormones, hormonal levels were measured using ELISA in hypothalamus homogenates. As shown in Figure 3A, the CRH levels of GF restraint-stressed mice exhibited a significantly greater increase than those of SPF restraint-stressed mice (*p* < 0.05). The concentrations of ACTH in GF restraint-stressed mice homogenates were higher than in the GF control group (*p* < 0.01) and SPF restraint-stressed mice (*p* < 0.01; Figure 3B). The results revealed a trend toward increased CORT concentration in the GF restraint-stressed group compared with the SPF restraint-stressed group (*p* < 0.05; Figure 3C). ALD levels in GF restraint-stressed mice were also increased in hypothalamus homogenates compared with GF control group (*p* < 0.05) and SPF restraint-stressed mice (*p* < 0.01; Figure 3D).

### Changes in hormone receptor mRNA

**Figure 4:**
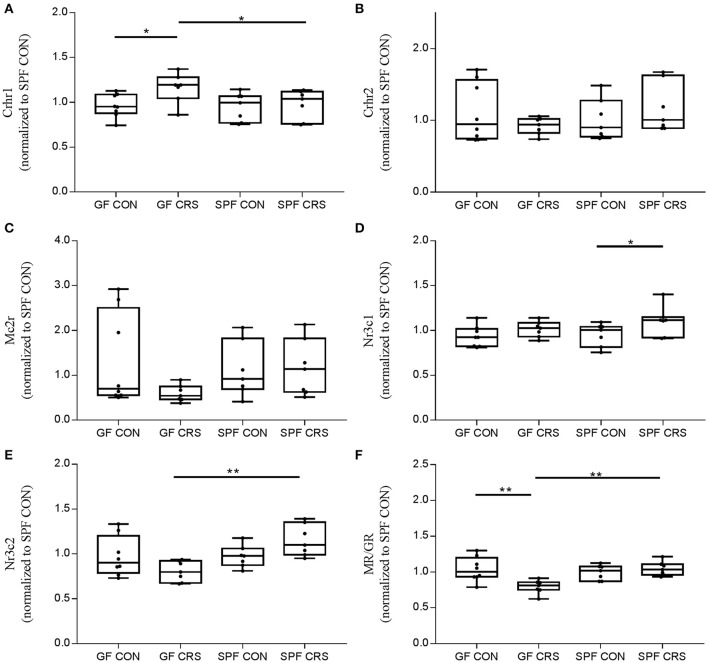
Validation of hormone receptors and mineralocorticoid receptor (MR) / glucocorticoid receptor (GR) expression changes in the hypothalamus. Expression of hormone receptors and MR/GR was assessed in GF mice, GF CRS mice, SPF mice, and SPF CRS mice (*n* = 7–8 in each group). The data were analyzed using two-way ANOVA. Crhr1, corticotropin releasing hormone receptor 1, CRFR1; Crhr2, corticotropin releasing hormone receptor 2, CRFR2; Mc2r, melanocortin 2 receptor, ACTHR; Nr3c1, nuclear receptor subfamily 3, group C, member 1, glucocorticoid receptor, GR; Nr3c2, nuclear receptor subfamily 3, group C, member 2, mineralocorticoid receptor, MR. **(A)** shows the hormone receptor change in HPA axis. [**A**, *F*_(3, 25)_ = 0.315; **B**, *F*_(3, 25)_ = 5.012;**C**, *F*_(3, 25)_ = 5.005; **D**, *F*_(3, 25)_ = 0.373; **E**, *F*_(3, 25)_ = 1.813]. The MR/GR expression ratio was calculated to assess receptor disorder [*F*_(3, 25)_ = 1.711, **F**]. The values were presented as single data points superimposed to boxplots ^*^p < 0.05, ^**^p < 0.01. All data were normalized to SPF control mice.

To investigate the link between hormone levels and hormone receptor mRNA, receptor mRNA in the mouse hypothalamus was quantified using qRT-PCR. Figure 4 shows the changes in receptor levels among the groups. As expected, the Crhr1 mRNA levels of GF CRS mice were increased compared with GF control (*p* < 0.05) and SPF CRS mice (*p* < 0.05; Figure 4A). However, Nr3c1 mRNA expression in SPF control mice was decreased compared with SPF CRS mice (*p* < 0.05; Figure 4D), and Nr3c2 mRNA expression was decreased in GF CRS mice compared with SPF CRS mice (*p* < 0.01; Figure 4E). de Kloet (2014) demonstrated that the MR/GR balance plays an important role in mediating the function of CORT in the brain, and that dysfunction of MR/GR expression can occur in specific pathological, emotional, and cognitive conditions (Brinks et al., 2007). To detect whether MR/GR expression had changed, we calculated the ratio of MR to GR and found that MR/GR decreased in GF CRS mice compared with GF control mice (*p* < 0.01). A decrease was also found in SPF CRS mice (*p* < 0.01; Figure 4F).

## Discussion

In our study, behavioral tests showed that GF control mice exhibited an increase in the distance traveled and time spent in the center of the OFT compared with SPF control mice, consistent with previous reports (Zeng et al., 2016; Zheng et al., 2016b). The GF mice without non-intestinal microbial colonization moved a greater total distance in the OFT and spent more time in the center, compared with SPF mice with intestinal microbes after CRS. This finding indicates that SPF mice with intestinal microbes exhibited increased anxiety-like behavior under the same pressure. Previous studies demonstrated that GF F344 rats were more likely to exhibit anxiety-like behavior than SPF rats (Crumeyrolle-Arias et al., 2014; Desbonnet et al., 2014; Wong et al., 2016; Zheng et al., 2016a,b). However, some studies found no relationship between intestinal microbes and animal behavior. The effects of intestinal microbes and physiological state on psychopathology are still debated. We then found that behavioral changes were largely consistent with changes in hormones, both in the presence of intestinal microbes and non-intestinal microbes. In addition, the results showed that hormones in GF CRS mice were significantly upregulated compared with SPF CRS mice in the HPA axis, in accord with previous reports (Sudo et al., 2004). This mechanism may be related to changes in CRH-signaling, glucocorticoids, or GR, which mediate behavior in the central nervous system (Owens and Nemeroff, 1991).

Although, some previous studies reported that anxiety- and trauma-related disorders were not consistent with simultaneous changes in the HPA axis, it is well established that these disorders are associated with an imbalance in the HPA axis (Smith et al., 1989; Baker et al., 1999; Jacobson, 2014). The current results revealed that GF CRS mice exhibited anti-anxiety behavior accompanied by HPA axis over-activity compared with SPF CRS mice. This novel finding may be related to our use of hypothalamus tissue, whereas many previous studies used plasma. The HPA axis has a complex feedback mechanism, and intestinal microbes may regulate behavior through the endocrine system, which may subsequently induce overactivity in the HPA axis.

Our examination of hormone receptors also indicated hormone dysfunction in the hypothalamus. Previous studies reported that hormone receptor gene knockout mice and mice given hormone receptor antagonists exhibited modulation of stress-coping behaviors (Boyle et al., 2005). GR is widely expressed in most cell types throughout the body (De Kloet et al., 2000). GR and MR act as ligand-activated transcription factors and affect gene transcription, playing an important role in glucocorticoid function (Reul and Kloet, 1985). In addition, researchers have reported that changes of GR or MR levels in the hippocampus are associated with HPA axis dysfunction in mood-related illness, although findings have been inconsistent, with some studies finding that GR mRNA is decreased in depression, and other studies reporting that GR mRNA in the hippocampus was unchanged (Webster et al., 2002). At the same time, down-regulated MR and GR expression, and changes in MR/GR ratio have been reported in stress-induced rats (Medina et al., 2013). In the current study, we also used the MR/GR expression ratio to assess receptor diversification in the hypothalamus after behavioral changes. The results revealed changes in MR/GR expression and the action of intestinal microbes.

Intestinal microbes constitute a large and complex ecosystem in the intestinal wall of animals, affecting physiological and neuronal function, as well as animal behavior, via the microbiota-gut-brain axis and metabolites. Taken together with the behavioral and hormonal variations described above, the current results indicate that intestinal microbes play a critical role in influencing behavior and HPA-axis regulatory imbalance under external stress. Recent research suggests that intestinal microbes affect the host's physiology, metabolism and immunology, as well as nervous system development and brain function, through the microbiota-brain-gut axis (Collins and Bercik, 2009; Fu et al., 2015; Yano et al., 2015). Interestingly, Bercik et al. (2011) reported that adult mice given microbial agents via oral absorption showed changes in exploratory behavior and brain-derived neurotrophic factor (BDNF) expression in the hippocampus, while no change was observed with intraperitoneal injection of the same agent.

**Figure 5:**
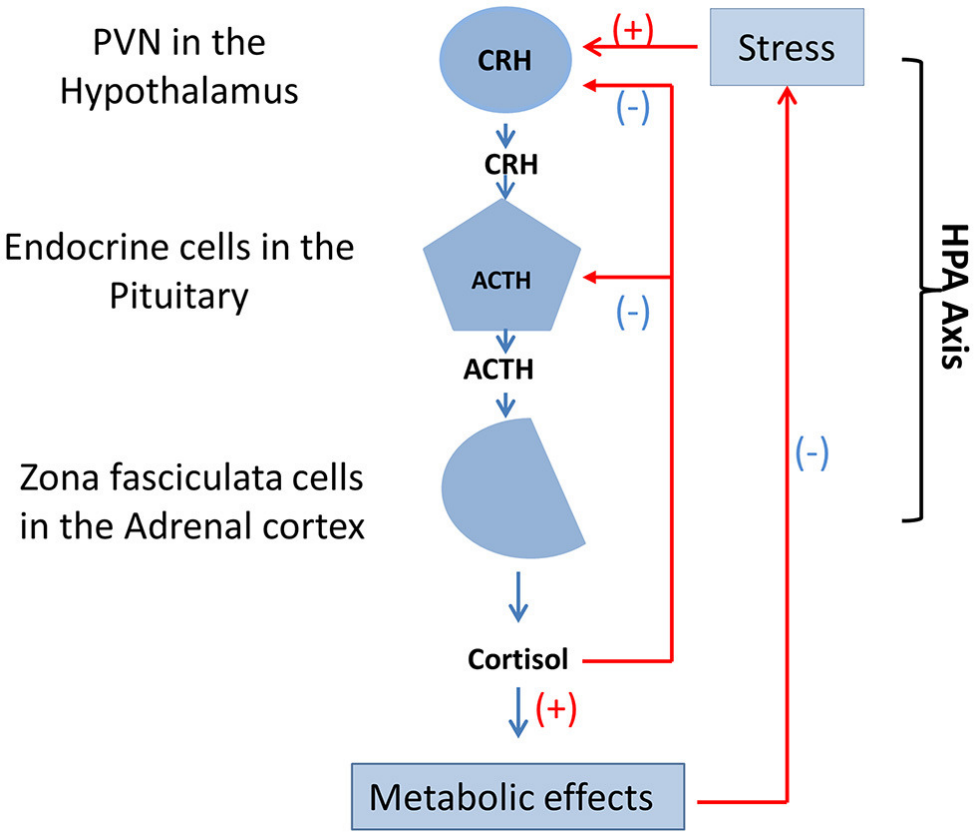
Function and mutual adjustment of HPA axis. The HPA axis contains three cell types that secrete three different hormones: neurons of the PVN in the hypothalamus secrete CRH, endocrine cells in the pituitary secrete ACTH, and zona fasciculata cells in the adrenal cortex secrete cortisol. Stress, drugs, and diseases produce positive feedback regulation of neurons in the of the medial parvocellular portion of the PVN. In addition, cortisol that could result in metabolic effects produces direct negative feedback suppression of endocrine cells in the pituitary and CRH neurons of the PVN in the hypothalamus, respectively. HPA axis, hypothalamic-pituitary-adrenal axis; PVN, hypothalamic paraventricular nucleus; CRH, corticotropin-releasing hormone; ACTH, adrenocorticotropic hormone.

Studies have also reported that high intestinal permeability, bacterial translocation, and inflammatory factors are an important factor in mental disorders. Intestinal microflora mediate a series of neurotrophic factors, BDNF, and proteins (Ait-Belgnaoui et al., 2005). Intestinal microbial immune disorders are associated with aberrant neurodevelopment, and inappropriate use of antibiotics inhibits short-chain-fatty-acids (SCFAs) and the interaction between toll-like receptors and Treg cells. Moreover, the HPA axis (Figure 5) is affected by the peripheral nervous system (PNS), infection, and stress. The proportion of carbohydrates in food and dietary structure can also affect HPA axis activity (Keating et al., 2004; Glover et al., 2010; Ronald et al., 2010; Smith et al., 2013).

In addition, we speculated that intestinal microbes might cause intestinal metabolic changes through the intestinal microbial-gut-brain axis pathway. Metabolites may then pass through the intestinal wall, into blood circulation and through the blood-brain-barrier (BBB). The central nervous system (CNS) may then be affected by products of bacterial metabolism, causing hormone and receptor dysfunction, as well as behavioral changes.

First, intestinal microbes through enterochromaffin (EC) cells control the synthesis of 5-HT, which could be involved in brain function (Yano et al., 2015). Second, microbes may have an important relationship with the CNS through the inflammatory pathway, possibly activating local or systemic immune responses through the vagus nerve to influence the activity of the brain-gut axis (Borovikova et al., 2000; Wang et al., 2003). Third, SCFAs produced by intestinal bacterial fermentation have an immunomodulatory function, stimulating the link between the sympathetic nerve and nerve cells through G-protein-coupled receptor 41 (GPR41) and 43 (GPR43; Kimura et al., 2011). This might regulate the balance of microgliacytes, and mediate the release of intestinal peptide from endocrine cells to affect brain-gut axis activity (Wren and Bloom, 2007). In addition, this may mediate 5-HT synthesis in EC cells, which provides the CNS termination signal (Yano et al., 2015). Finally, intestinal microbes regulate tryptophan metabolism, which affects brain function and plays an important role in serotonin synthesis in the CNS (Ben-Ari, 2013). Moreover, intestinal microbes may also produce dopamine, γ-aminobutyric acid, histamine and acetylcholine, regulating the function of CNS and the stability of the HPA axis (Thomas et al., 2012; Barrett et al., 2014). In accord with this notion, the microbiota-gut-brain axis is considered to function as a bidirectional regulation mechanism of animal behavior (Wong et al., 2016).

The current study involved several limitations that should be considered. First, we did not use multiple behavioral paradigms to examine behavior more comprehensively. Moreover, this experiment did not clarify which intestinal microbial flora induced behavioral and endocrine changes in mice. Thus, more in-depth examination of the possible mechanisms involved should be conducted in follow-up research. In addition, in future studies we plan to re-colonize known microorganisms or probiotics into the intestine to regulate the connection between the intestine and the brain in mice, then utilize the corresponding intestinal microbe antibiotics, hormone or receptor antagonists to interfere with the connection, to further reveal the functional mechanisms of microorganisms in the HPA axis.

## Conclusions

Based on previous research, in the current study we predicted that intestinal microbes would be an important factor in balancing the HPA axis. Imbalances of the HPA axis caused by intestinal microbes can affect the neuroendocrine system in the brain, resulting in an anxiety-like behavioral phenotype. The current findings suggest the possibility that novel treatments could be developed for stress-related diseases, including anxiety disorders, by direct or indirect intervention in intestinal microbial flora with currently available drug treatments.

## Author contributions

RH, BZ, BL, YL, HyW, CZ, LF, WL, and RN: Performed experiments; LZ, RH, PX, and HW: Designed the study; RH and KC: Wrote the manuscript; All authors reviewed and approved the manuscript prior to its submission.

### Conflict of interest statement

The authors declare that the research was conducted in the absence of any commercial or financial relationships that could be construed as a potential conflict of interest.
